# Patterns and Temporal Dynamics of Natural Recombination in Noroviruses

**DOI:** 10.3390/v15020372

**Published:** 2023-01-28

**Authors:** Yulia A. Vakulenko, Artem V. Orlov, Alexander N. Lukashev

**Affiliations:** 1 Martsinovsky Institute of Medical Parasitology, Tropical and Vector Borne Diseases, Sechenov First Moscow State Medical University, 119435 Moscow, Russia; 2 Faculty of Bioengineering and Bioinformatics, Lomonosov Moscow State University, 119234 Moscow, Russia; 3 Research Institute for Systems Biology and Medicine, 117246 Moscow, Russia

**Keywords:** norovirus, recombination, modular evolution

## Abstract

Noroviruses infect a wide range of mammals and are the major cause of gastroenteritis in humans. Recombination at the junction of ORF1 encoding nonstructural proteins and ORF2 encoding major capsid protein VP1 is a well-known feature of noroviruses. Using all available complete norovirus sequences, we systematically analyzed patterns of natural recombination in the genus *Norovirus* both throughout the genome and across the genogroups. Recombination events between nonstructural (ORF1) and structural genomic regions (ORF2 and ORF3) were found in all analyzed genogroups of noroviruses, although recombination was most prominent between members of GII, the most common genogroup that infects humans. The half-life times of recombinant forms (clades without evidence of recombination) of human GI and GII noroviruses were 10.4 and 8.4–11.3 years, respectively. There was evidence of many recent recombination events, and most noroviruses that differed by more than 18% of nucleotide sequence were recombinant relative to each other. However, there were no distinct recombination events between viruses that differed by over 42% in ORF2/3, consistent with the absence of systematic recombination between different genogroups. The few inter-genogroup recombination events most likely occurred between ancient viruses before they diverged into contemporary genogroups. The recombination events within ORF1 or between ORF2/3 were generally rare. Thus, noroviruses routinely exchange full structural and nonstructural blocks of the genome, providing a modular evolution.

## 1. Introduction

Noroviruses are small non-enveloped single-stranded positive-sense RNA viruses that comprise the genus Norovirus in the family *Caliciviridae*. Noroviruses infect a wide range of mammalian species. Human noroviruses are the major cause of sporadic and epidemic acute gastroenteritis in people of all ages [1].

Noroviruses have a 7.5–7.9 kb long RNA genome with a VpG peptide covalently linked to the 5′end, and a polyadenylated 3′end. The genome of most noroviruses is organized into three open reading frames (ORFs), while in murine noroviruses the fourth ORF that encodes a virulence factor was described. ORF1 encodes a polyprotein that is co- and post-translationally cleaved into six nonstructural proteins (i.e., not included in the capsid). ORF2 overlaps by 14–20 nt with ORF1 and is generally translated from a subgenomic RNA [2], while bovine norovirus (genogroup GIII) uses translation termination/reinitiation as an additional mechanism to express ORF2 [3]. ORF2 encodes the major capsid protein VP1, which contains a shell domain (S) and a protruding domain (P). The P domain is divided into a moderately variable P1 stalk domain and an exposed hypervariable P2 domain. ORF3 encodes the minor capsid protein VP2 and overlaps by 1 nt with ORF2 [2].

Based on the diversity of VP1 sequence, noroviruses are divided into ten genogroups (GI–GX) and at least 49 genotypes [4]. Viruses of genogroups GI, GII, GIV, GVIII and GIX infect humans; three genotypes of GII infect pigs; GIII includes bovine and ovine noroviruses; GV viruses infect mice and rats; viruses from GIV and GVI infect both cats and dogs, while GVII consists of canine noroviruses; finally, GX comprises novel bat noroviruses [5]. To account for the recombination between ORF1 and ORF2, a dual nomenclature that uses both the RNA-dependent RNA polymerase (RdRp) (3′ terminal) region of ORF1 and the major capsid protein VP1 nucleotide sequences has been proposed. According to it, noroviruses are divided into eight confirmed and two tentative P-groups and 60 P-types based on the diversity of the RdRp region [6].

Recombination in noroviruses is widespread and is one of the driving forces of increasing norovirus diversity. First reports of natural recombination between GII viruses were based on analysis of sequences of circulating viruses [7,8]. Since then, recombinant noroviruses have been reported in many studies. Most events described in the field occurred at the ORF1/ORF2 junction. The recombinants belonged to GI-GV, and the vast majority of cases of recombination were within a genogroup (reviewed in [9]). To the best of our knowledge, only four intergenogroup recombination events were described between viruses from GI.P8 and GII.4 [10]; GII and GIV [11]; GIV and GVI [12]; GIV and GVI.2 [13]. In addition to the typical recombination point at the ORF1/ORF2 junction, a number of events were reported within the VP1 coding region with breakpoints in S/P1, P1-1/P2 junctions [14,15], within P1 [11] and within P2 [14,16]. There were also isolated reports of recombination within ORF1 [17,18] and at the ORF2/ORF3 junction [15,19,20] between GII, between GIV noroviruses, as well as recombination within ORF3 encoding the capsid protein VP2 in GV noroviruses [21,22]. The first in vitro experimental evidence of norovirus recombination was documented in murine noroviruses (GV) at the ORF1-ORF2 overlap [23]. Another study analyzed murine noroviruses from coinfected mice and detected frequent recombination at the ORF1/2 junction, and recombination events with low frequency in the VPg, protease and 3′end of the RdRp coding region, and in the S domain of VP1 [24].

Most recombination events were inferred between viruses infecting the same host species. Viruses from a few genogroups, namely GII, GIII, GIV, GV and GVI, can infect several host species. However, recombinants between viruses of different hosts are rare: one and only virus that was a possible recombinant of feline (GIV) and canine (GVI) noroviruses has been described to date [13,25].

Recombination in noroviruses has been well explored across the genome and among the genogroups [9]. However, the temporal dimension was lacking, likely because the number of known genomes has been limited. The extent of genetic diversity between noroviruses involved in recombination has been explored only vaguely, because common bioinformatics tools are primarily aimed at detection of recombination events, not at properties of putative parents and the impact of recombination on the taxon as a whole. In picornaviruses, which were historically better studied compared to noroviruses, there have been several estimates of the recombination frequency over time [26,27,28]. Moreover, ubiquitous recombination has been pointed out as a force shaping the species and is considered one of the species criteria [29]. It is plausible that the mechanisms and patterns known for picornaviruses would also be valid for noroviruses. In this study, we aimed to systematically analyze natural recombination in the genus *Norovirus* using all data available in public databases both throughout the genome and over time.

## 2. Materials and Methods

### 2.1. Dataset Preparation

Complete genome sequences available for the genus *Norovirus* (*n* = 3439) were downloaded from the Genbank database as of July 2020. Sequences with more than 1% ambiguous nucleotides or more than five ambiguous nucleotides in a row were omitted from the dataset. The remaining ambiguous nucleotides were automatically resolved to a consensus using a custom Python script available at https://github.com/v-julia/resolve_ambiguous (accessed on 27 November 2022). The coordinates of ORF1, ORF2 and ORF3 were extracted from GenBank annotations. Then, the nucleotide sequences of ORFs were excised from the full genome sequences and aligned separately based on their corresponding amino acid translations using mafft v7.450 [30]. Next, the resulting nucleotide alignments of ORFs were concatenated, and the columns containing more than 20% gaps were removed using trimAl [31]. Since the ORFs were concatenated, their overlapping regions (17 nt between ORF1 and ORF2, 1 nt between ORF2 and ORF3) were duplicated in alignments. Finally, sequences sharing more than 99.5% identity were excluded. The resulting alignment of concatenated ORFs contained 1084 nucleotide sequences. The virus host and collection date were retrieved from GenBank entries automatically using custom python script and manually verified for all sequences in the dataset. The genogroups, genotypes, P-groups and P-types of viruses were designated using “Norovirus Typing Tool version 2.0” [32]. The final alignment, as well as scripts used for alignment preparation and data retrieval are available at https://github.com/orlovartem/NoV_recombination (accessed on 27 November 2022).

### 2.2. Recombination Analysis

The preliminary analysis of recombination patterns was performed via the computation of phylogenetic compatibility matrices [33,34] implemented in RDP4 software [35]. Phylogenetic compatibility matrices allow us to observe the changes in phylogenetic relationships of sequences in different genome regions. The algorithm computes phylogenetic trees for sequentially generated regions of 600 nt in length, sliding in steps of 50 nt, and then calculates Robinson–Foulds distance (the minimum number of edge contraction and extension needed to transform one tree into the other) between them. Then, the resulting distance matrix was visualized as a heatmap. The full exploratory recombination analysis of sequences was performed using nine methods implemented in RDP4: RDP [36], GENECONV [37], Bootscan [38], Maxchi [39], Chimaera [40], SiSscan [41], PhylPro [42], LARD [43], 3Seq [44]. The events supported by at least four methods were considered in this study. This level of confidence was chosen arbitrarily, and thus, the number of recombination events from this test could not be interpreted quantitatively. RDP4 yields a potential recombinant sequence, its major and minor parents and coordinates of recombination breakpoints. The part of genome in the recombinant sequence obtained from a minor parent according to RDP4 is hereinafter referred to as a recombinant fragment. The coordinates of recombinant fragments and numbers of recombination breakpoints across the genome were visualized in RStudio (ggplot2 package [45]).

Additionally, we used another approach based on correlation of accumulation of substitutions in different genome regions [46]. First, pairwise distance correspondence (PDC) plots were computed to visualize recombination between specific genetic regions. For PDC plots, pairwise genetic distances are calculated for two genomic regions and then plotted. Each point of the plot corresponds to the genetic distances between two sequences. When recombination is absent, the pairwise genetic distances should generally follow a linear relationship. If sequences with recombination between two genomic regions are present in the dataset, then the distances between recombinant and “parental” sequences will diverge from the regression line. PDC plots using all even vs. all odd positions in an alignment were used as control to illustrate stochastic deviations from the regression line. PDC plots show the general distribution of pairwise distances and are viewed as a visualization method and an addition to RDP4 methods, because the result is not quantitative, and they do not provide statistical significance of the findings. The sensitivity of PDC plots depends on the length of genetic fragment considered. In simulated datasets, a 4% nucleotide sequence distance between the parental genomes was sufficient to identify the deviating dots corresponding to recombinants in alignments with length above 1500 nt (data not shown).

To illustrate the recombination patterns across the genome, we also computed pairwise distance deviation (PDD) matrices, which reflect the extent of sequence distance incongruence (putative recombination) between different genomic regions. To compute them, pairwise genetic distances are calculated for all genome regions of 600 nt in length sliding by a step of 50 nt. Then, for each pair of genomic regions, the linear regression model is built, and root-mean-square error (RMSE) is calculated. The RMSE reflects the sum of incongruences of pairwise genetic distances between two genomic regions. RMSEs for each possible pair of regions are visualized as a heatmap. Calculation of PDCPs and PDD matrices was implemented as the R package “recDplot” (https://github.com/v-julia/recDplot, accessed on 27 November 2022).

### 2.3. Phylogenetic Analysis

The phylogenetic trees of VP1 and RdRp-encoding nucleotide sequences were inferred using IQ-TREE v1.6.12 [47] with 10,000 pseudo-replicates [48], incorporating the best-fit model of nucleotide substitution (VP1: TIM2 + F + R10, RdRp: GTR + F + R10) [49], and rooted by a midpoint. Trees were visualized with ggtree R-package [50].

Maximum clade credibility (MCC) trees for sequences with available collection dates were inferred for GI (N = 71 sequences) and GII (N = 915 sequences) using BEAST v.1.10.4 [51]. The best-fit partitioning scheme (GI: (1 + 2)(3), GII: (1,2,3)) and substitution models (GTR + I + G + X) for Bayesian analysis were chosen according to the Bayesian Information Criterion using the PartitionFinder 2 program [52]. For each genogroup, marginal likelihoods were calculated for combinations of coalescent tree priors (coalescent constant size, coalescent exponential growth) and molecular clock models (strict, relaxed log-normal) using the path sampling/stepping stone procedure implemented in BEAST v1.10.4 [53]. Then, different model settings were compared using the Bayes factor (BF) test. The combination of coalescent constant prior and relaxed lognormal molecular clock was strongly favored (log BF > 10) for both genogroups. The MCMC chains were run for 50 and 800 million steps with sampling every 5000 and 10,000 steps for GI и GII, respectively. The convergence of Markov chain Monte Carlo (MCMC) was inspected using Tracer v1.7 [54]. The maximum clade credibility (MCC) tree was annotated with TreeAnnotator v1.10.4 using 10% burn-in.

### 2.4. Calculation of Recombination Half-Lives

To calculate the half-lives of recombinant forms (RFs) in GI (N = 71 sequences) and GII (N = 915), two approaches were applied. In the first approach [26,28], the distances were calculated using the Maximum Composite Likelihood approach (MCL distances) for sequence pairs with the same VP1 genotype in MEGA v.7 [55]. Then, the proportions of comparisons where viruses had the same VP1 genotype but different P-types were calculated for ranges of MCL distances. Here, such comparisons of viruses with coinciding VP1 genotypes and different P-types are referred to as RFs. The MCL distance corresponding to 50% RFs among all pairwise comparisons was calculated using linear regression implemented in sklearn [56]. To calculate the half-life of RF, the following formula used in a number of previous studies was applied [26,27,28]:

RF half-life = MCL distance/(rate × 2)(1)

where ‘MCL distance’ is the threshold MCL distance where 50% of sequence comparisons were recombinant, and ‘rate’ is the substitution rate in the VP1 region inferred using BEAST software (see Phylogenetic analysis section). The division of the threshold MCL distance by substitution rate corresponds to the combined time of divergent evolution of two genomes. The division by 2 is needed to calculate the period of divergence of two contemporary sequences from a common ancestor. In GI, recombination events were less common than in GII, and involved the change in topology of the whole clades, which led to change of the capsid genotype of the recombinant virus. Therefore, this approach was not suitable for the calculation of RF half-life for GI.

In the second approach [57], clades with non-recombinant viruses were identified manually for MCC trees for RdRp and VP1 of GI and GII inferred in BEAST software and their median ages were determined. Only clades with posterior probabilities greater than 0.9 in both genomic regions were used for the calculation of RFs half-lives.

## 3. Results

### 3.1. General Patterns of Recombination in Noroviruses

**Figure 1:**
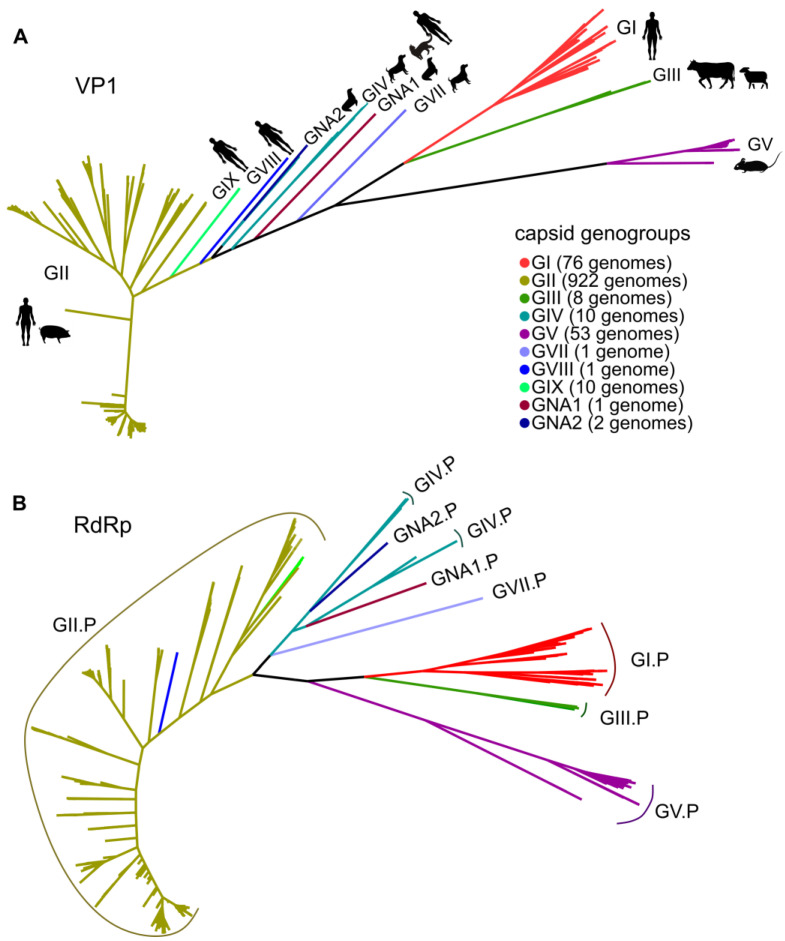
The maximum likelihood tree of the VP1 (**A**) and RdRp encoding (**B**) sequences (N = 1084) of the genus *Norovirus*. The tree branches are colored according to the capsid genogroup in both trees. The number of sequences in the dataset belonging to each capsid genogroup is indicated in brackets. In the phylogenetic tree for RdRp P-groups are shown. GII.P includes viruses with GII, GVIII and GIX capsid genogroups. The silhouettes of hosts infected by norovirus genogroups are shown near the tips.

To investigate the extent of intra- and inter-genogroup recombination in noroviruses, we obtained all complete nucleotide sequences of norovirus genomes available in Genbank. After removing nearly identical sequences, the final dataset contained 1084 full genomes. The sample of sequences for different genogroups was uneven (Figure 1). Most norovirus sequences (922 out of 1084) belonged to GII, which infects humans. More than half (567) of GII sequences belonged to the genotype GII.4, which has been the cause of most norovirus gastroenteritis outbreaks. To account for a possible sample bias, two separate analyses were conducted for GII and the remaining genogroups.

**Figure 2:**
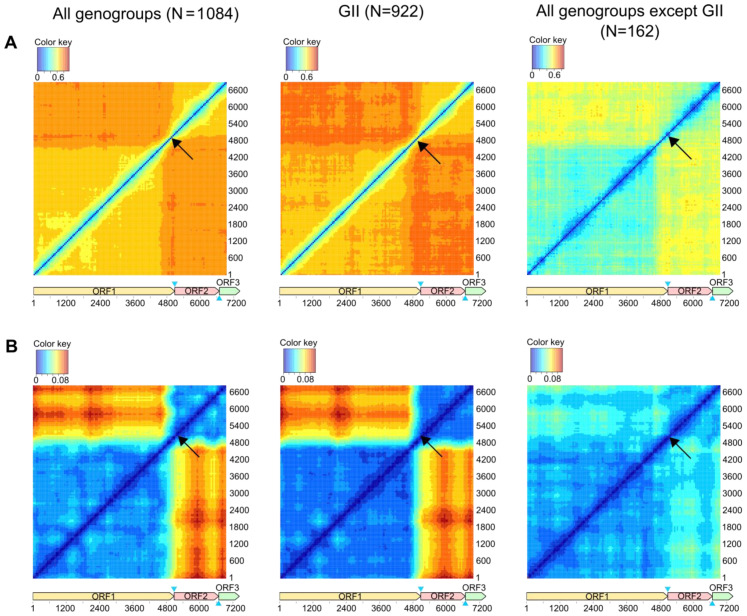
The recombination in ORF1/2 junction is the most prominent in all genogroups of noroviruses, but can be suggested elsewhere in the genome. Recombination incidence in norovirus genome detected by phylogenetic compatibility matrices (**A**) and pairwise distance deviation (PDD) matrices (**B**) for noroviruses with the following parameters: window = 600 nt, step = 50 nt. Axes indicate the position of a window start in the alignment. Colors reflect normalized Robinson–Foulds distances (**A**) and root-mean-square error (RMSE) in pairwise distance correspondence (PDC) plots built for the corresponding alignment window pairs (**B**). Blue triangles indicate the overlaps between ORF1 and ORF2 (17 nt), ORF2 and ORF3 (1 nt) that were duplicated in the alignment of concatenated ORFs. Black arrows indicate the ORF1/2 junction.

First, we used genome-scale recombination analysis tools to infer the global patterns of genome regions exchange. The phylogenetic compatibility matrix showed that phylogenetic trees produced from ORF1 were the most incompatible with the ORF2-ORF3 (Figure 2), confirming a well-known recombination hot spot (arrows on Figure 2). Although the phylogenetic compatibility within ORF1 and ORF2-3 was higher than between them, the phylogenetic trees within these regions were also somewhat discordant. The pairwise distance deviation matrices were generally consistent with the phylogenetic compatibility matrices (Figure 2B). Apparently, the recombination patterns throughout the genome were not altered by many recent sequences that were added to the norovirus dataset compared to previous studies. Recombination profile throughout the genome was remarkably similar between GII and other genogroups, despite differences in absolute values of Robinson–Foulds distances and root-mean-square error values due to dissimilarity of the datasets. Unfortunately, the output of these methods could not be used to compare the frequency of recombination in GII and other genogroups, because the size, composition, and diversity of datasets, and lack of reliable approaches to normalize these values.

### 3.2. Recombination Breakpoints throughout the Genome and among Genogroups

**Figure 3:**
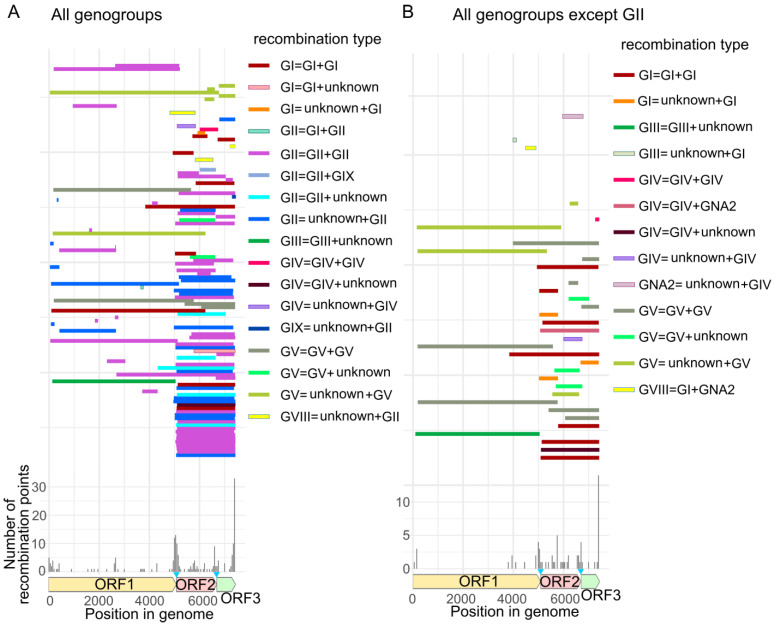
The distribution of recombinant fragments detected by at least four algorithms implemented in RDP4 in two datasets: (**A**)—the dataset that includes all norovirus genogroups; (**B**)—the dataset with all genogroups except GII. Blue triangles indicate the overlaps between ORF1 and ORF2 (17 nt), ORF2 and ORF3 (1 nt) that were duplicated in alignments of concatenated ORFs. Recombination type legend refers to the VP1 genogroups of a recombinant norovirus, and its minor and major parents (recombinant = minor parent + major parent). The lower panel shows the number of recombination breakpoints detected by RDP4 at each position of the alignment.

Since the phylogenetic compatibility matrices indicate only relative abundance of recombination across the genome and do not show the particular sequences involved in recombination, we proceeded with an exploratory analysis using all available tools in RDP4. Most recombinant fragments constituted almost full ORF2 + ORF3, and such recombination events with a break point near the ORF1/ORF2 junction were found in all genogroups (Figure 3). However, there were also smaller recombinant fragments from 55 nt to 1500 nt within all ORFs. Recombination events within the structural genome region were somewhat more abundant at the ORF2/ORF3 junction, but individual breakpoints were also mapped within VP1 (GI, GII and GV) or VP2 (GI, GII, GIV and GV). Ends of recombinant fragments mapping near genome termini could be analysis artefacts.

Most recombinants were a result of intragenogroup events, and all but one of them involved viruses from a same host. The exclusion included recombinant GIV viruses from cats and dogs that had VP1 region most close to human norovirus. In addition, several intergenogroup recombinants were also detected (Figure 3, Table S1). Most intergenogroup recombinants supported by at least four algorithms in RDP4 were found among human viruses (genogroups GII, GI, GVIII and GIX). There were only two intergenogroup recombination events at ORF1/ORF2 junction. Particularly, the fact that GVIII are a result of recombination of GII noroviruses and unknown noroviruses was supported by different grouping of GVIII strains on the phylogenetic trees of VP1 and RdRp. Most intergenogroup recombination events had breakpoints within ORFs (Table S1). The viruses involved in intergenogroup recombination differed by 30–50% of nucleotide sequence in both genomic regions. For example, recombinant GIV norovirus differed by 35% from GIV noroviruses and by 45% from GNA2 noroviruses in ORF2-ORF3 region. Thus, the observed intergenogroup recombination events were not recent and could have occurred before viruses diverged into distinct genogroups. Therefore, they were not necessarily “intergenogroup” at the time they happened.

### 3.3. Genetic Divergence of Noroviruses Involved in Recombination

Although the tools from RDP4 locate the recombination events, they do not indicate whether these events occurred recently among closely related viruses or not. To get an overview of the extent of genetic divergence of recombinant sequences, we built pairwise distance comparison (PDC) plots that visualize the relationship of genetic distances in two genomic regions. When recombination is absent, genetic distances correlate because the substitutions in two genomic regions accumulate proportionally. A single recombination event would result in deviation of several points from the regression line, which correspond to pairwise comparisons of a recombinant to its major and minor parents and their close relatives. A gene transfer between ancestral viruses would lead to cloud-like groups of points because such events are reflected in many descendant sequences.

**Figure 4:**
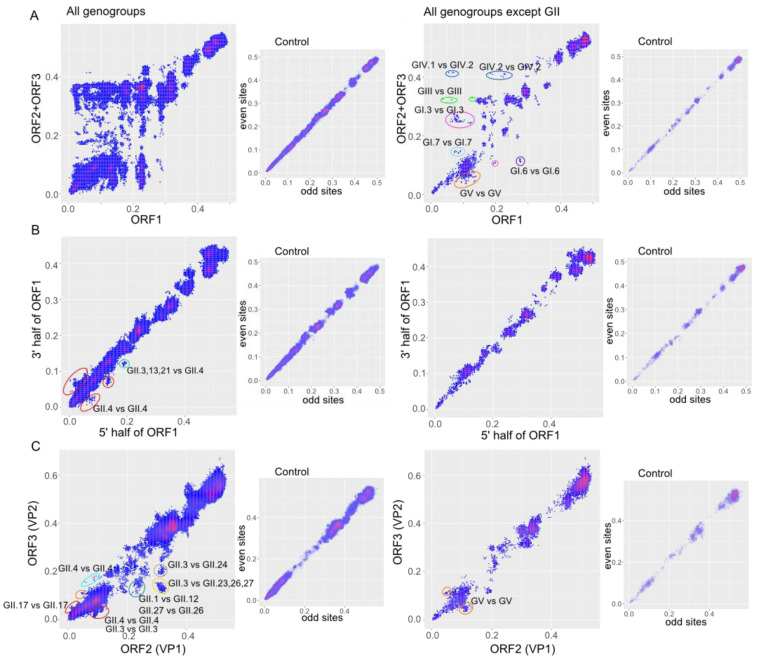
Correspondence between p-distances (PDC plots) between nonstructural (ORF1) and structural (ORF2, ORF3) genomic regions (**A**), within nonstructural genomic region (ORF1) (**B**), within structural genomic region (**C**) in all norovirus genogroups (left panel) and all genogroups except GII (right panel). The axes represent uncorrected p-distances in genomic regions considered. Control plots of correspondence between distances in even vs. odd positions in an alignment simulate PDC plots in absence of recombinant sequences in a dataset. Dots that correspond to virus pairs that underwent recombination and are discussed in the text are marked with circles.

PDC plots of nonstructural (ORF1) and structural (ORF2, ORF3) genome regions showed the most prominent disparity of pairwise distances, compatible with numerous recombination events suggested by other methods (Figure 4A). Pairwise genetic distances between viruses belonging to GII formed a symmetrical cloud within up to 40% of the nucleotide distance (Figure 4A). Virus pairs whose distances deviated from linear relationship represented different genotypes of GII. In GII, there was evidence of recent recombination events between viruses differed by just a few percent of nucleotide distance in one of the genomic regions. At an inferred substitution rate of 4.34 × 10^−3^ substitutions/site/year (s/s/y) (see below), one percent of genetic distance corresponds roughly to 2.3 years. In other genogroups, recombination was also notable. Recombination involved both distantly related viruses with nucleotide sequences ranging from 20% to 40% (representatives of genogroups GI, GIII, GIV) and moderately related viruses with nucleotide distances of less than 20% (GV, GI) (Figure 4A). Thus, the structural and nonstructural genomic regions of rather divergent noroviruses are fully compatible within a genogroup without any sequence distance-related restrictions, and co-infection of the same cell by distinct viruses happened frequently enough for these multiple recombination events to occur.

Both phylogenetic compatibility matrices, PDC plots and RDP4 analysis suggested moderate level of recombination in GII and a low level of recombination in other genogroups within the nonstructural genes (ORF1). Indeed, PDC plots detected obviously recombinant sequences only from GII, which is consistent with the results of RDP4. There were very recent recombination events between viruses of the same VP1 and P-type (GII.4 VP1 type and GII.P4 P-type) (Figure 4B, red circles). Additionally, there was a suggestive recombination event that was reflected in several sequences of GII.3_GII.P21, GII.13_GII.P21 and GII.21_GII.P21 types as one partner, and GII.4_GII.P31 as the other one (Figure 4B, green circle). Unfortunately, it is not possible to reconstruct exact recombination partners in this case, and it is even possible that this event occurred before these viruses diverged into distinct P-types.

Recombination between VP1 and VP2 was found in viruses of GII, GV and GI (Figure 4C). In line with data from other methods, several events could be suggested, more than within ORF1, but less than between ORF1/ORF2. Unlike recombination between ORF1 and ORF2, which maps almost precisely to the ORF1-2 junction, recombination between ORF2 and ORF3 could occur anywhere within these ORFs (Figure 3), and here ORF2 and ORF3 were analyzed just as an example. Almost all recombination events identified using PDC plot coincided with the results of the RDP4 analysis. Recombination between ORF2 and ORF3 involved both genetically close viruses with distances of less than 3% (Figure 4C, red circles) and more divergent viruses with genetic distances up to 20–35% (Figure 4C, dark green, yellow, gray circles). Almost all incongruences between ORF2/3 but one involved GII viruses, but this might reflect sample bias towards GII in the dataset.

### 3.4. Temporal Aspects of Norovirus Recombination

**Figure 5:**
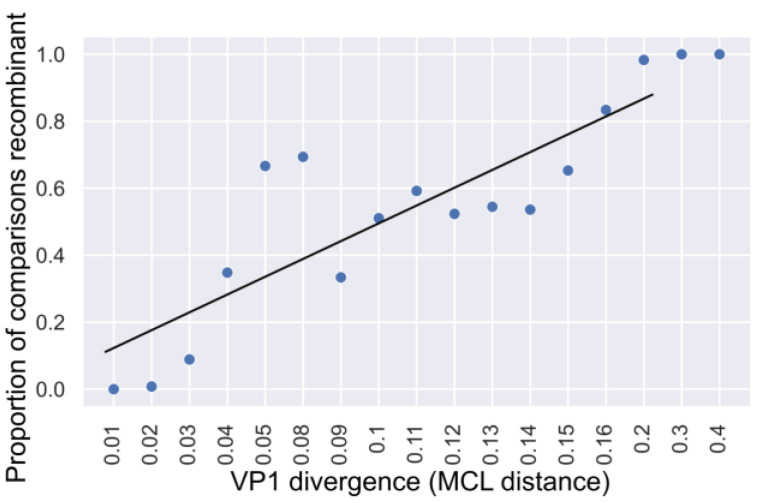
Association between pairwise VP1 gene nucleotide sequence divergence (x axis shows mean distance in each category) and proportion of recombinant comparisons (y axis) for GII noroviruses.

Genetic distances provide only a rough image of the temporal dynamics of recombination. Understanding temporal dynamics of recombination might give the new insights of molecular epidemiology of human noroviruses. Two genogroups that cause gastroenteritis outbreaks in human, GI and GII, had sufficient sequences for a more detailed analysis of recombination temporal dynamics. To evaluate the half-life of RF in the genogroup GII, the relationship between VP1 sequence divergence and the likelihood of recombination was determined, as was performed previously for enteroviruses [26,27,28]. For pairwise comparisons of isolates with the same VP1 genotype within genogroup GII, the MCL distance of VP1 genes was recorded for virus pairs of the same or different P-type. Genomes with the same VP1 genotype, but different P-types, were assumed to be recombinant. The proportion of comparisons where isolates had different P-types was raising with the increase of VP1 divergence, reaching 100% at MCL distances over 0.2 (Figure 5). A linear regression suggested that the MCL distance of 0.0982 corresponded to 50% of recombinant forms (a combination of distinct VP1 genotypes and P-types). Using the substitution rate in VP1 region inferred in Bayesian phylogenetic analysis, the time period needed to achieve this divergence was calculated (0.0982/4.34 × 10^−3^) that corresponds to 11.3 years of divergent evolution from the common ancestor. The inferred half-life of norovirus GII RF was thus 11.3 years. It should be noted that this method accounts only for recombination events at the ORF1/2 junction and does not consider intragenotype recombination. Additionally, it disregards recombination between viruses of the same VP1 genotype and P-type. Thus, it can be viewed as a conservative estimate.

**Figure 6:**
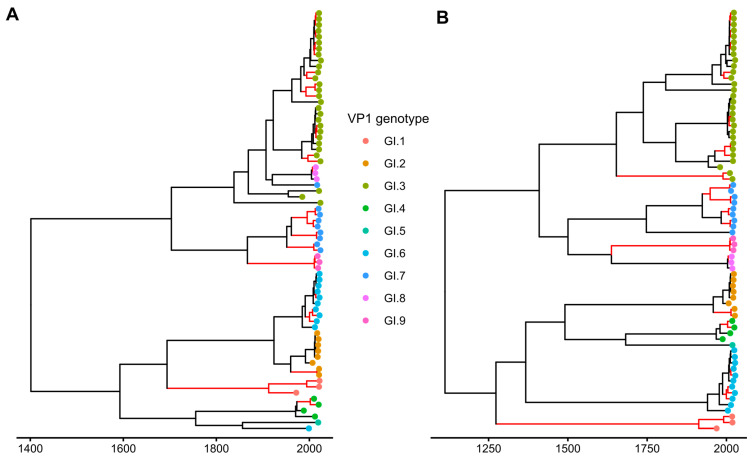
MCC trees of RdRp (**A**) and VP1 (**B**) genes of GI noroviruses. The scale bar represents time in years. Branches leading to matching subtrees in both trees are colored red. Since all branches in both trees had posterior probabilities above 0.92, branch support is not indicated on the figure. The tree branches are colored according to the VP1 genotype.

**Figure 7:**
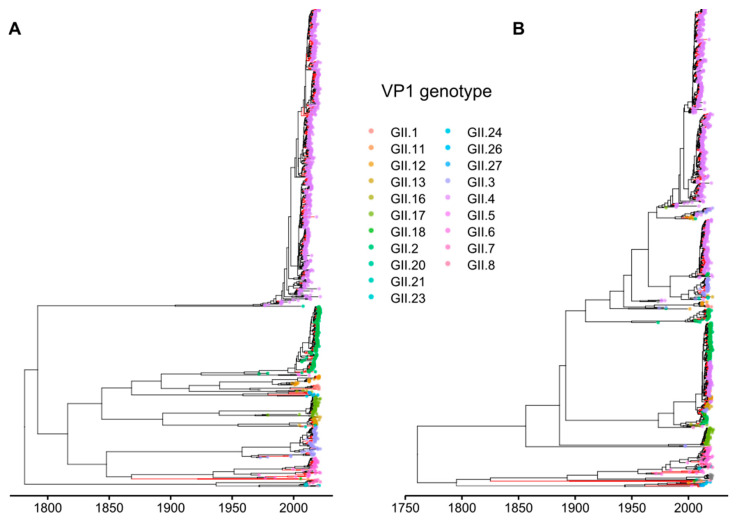
MCC trees of RdRp (**A**) and VP1 (**B**) genes on GII noroviruses. The scale bar represents time. Branches leading to matching subtrees in both trees are colored red. Bootstrap support was not indicated due to graphical considerations. The tree branches are colored according to the VP1 genotype.

In GI, most recombination events led to the change of position of the whole clades in the tree. Since the previous method did not take this into account, RF half-life was determined using an alternative approach [57] as the median age of nodes that led to clades with non-recombinant viruses (with identical grouping in RdRp and VP1), and was 10.44 years (Figure 6). The RF half-life of GII calculated using this approach was 8.64 years, which is consistent with the results of the first method (Figure 7). It is noteworthy that the overall parameters of the Bayesian phylogenetic inference were compatible with previous studies. The median time of the most recent common ancestors (tMRCAs) for GI noroviruses RdRp and VP1 genes dated back to 637 and 952 years ago, respectively, with overlapping 95% highest posterior density (HPD) intervals ([403–917] and [511–1483] years). The inferred substitution rates in RdRp and VP1 were 1.55 × 10^−3^ s/s/y [95% HPDs 1.03 × 10^−3^–2.05 × 10^−3^] and 1.41 × 10^−3^ s/s/y [8.57 × 10^−4^–2.06 × 10^−3^], respectively. This is in correspondence with the results of Bayesian phylogenetic analysis in [58,59], where tMRCA of norovirus GI strains were shown to diverge about 750 years ago, and the inferred substitution rates were in the range between 1 × 10^−3^–2 × 10^−3^ s/s/y. The MRCAs of GII RdRp and VP1 genes existed 245 [179–326] and 264 [198–347] years ago, and the substitution rates were 3.41 × 10^−3^ s/s/y [3.13 × 10^−3^–3.69 × 10^−3^] and 4.23 × 10^−3^ s/s/y [3.88 × 10^−3^–4.59 × 10^−3^]. This is also in line with earlier studies [59,60].

## 4. Discussion

Frequent recombination at the junction of ORF1 encoding nonstructural proteins and ORF2 encoding the major VP1 capsid protein is a characteristic feature of noroviruses [9,61]. The dual nomenclature based on the RdRp region of ORF1 and ORF2 that accounts for recombination is routinely used for norovirus typing worldwide [4,6] and reflects this phenomenon. A recent comprehensive review of norovirus sequences from public databases showed that more than half of available sequences were recombinants in the ORF1/2 junction [62]. In line with previous reports, both phylogenetic compatibility matrices and PDD matrices for noroviruses showed a pronounced recombination hot-spot at the ORF1/2 junction, indicating that phylogenetic trees and pairwise genetic distances built using ORF1 were the least compatible with the ORF2-ORF3 region (Figure 2). A full-scale analysis of norovirus sequences by all recombination detection algorithms implemented in RDP4 also showed that the majority of recombinant fragments were almost complete VP1-VP2 encoding regions (Figure 3).

Recombination events between nonstructural genomic region (ORF1) and structural genomic region (ORF2 and ORF3) were found in all analyzed genogroups of noroviruses, although recombination was most prominent between members of GII that infect humans. However, this could be a sample bias artifact because different GII genotypes are responsible for most gastroenteritis outbreaks in humans [63] and hence are more often sequenced. Dozens of cases of intergenotypic recombination between GII viruses have been described in the literature [9], and intergenotypic recombination is believed to be key factor in the generation of new norovirus variants that substitute older lineages in circulation. Pairwise genetic distances between GII noroviruses formed a symmetrical cloud within up to 40% difference in the nucleotide sequence on the PDC plots, which is compatible with multiple recombination events (Figure 4A). There were many virus pairs with distances that differed by only a few percent in one of the genomic regions and up to 40% in another one, suggesting that many recombination events occurred very recently. The half-lives of RF of human GI and GII noroviruses were calculated in this study by two approaches used previously for enteroviruses [26,28,57] and were 10.4 and 11.3/8.64 years, respectively. Thus, RF half-lives in human noroviruses were greater than those of enteroviruses (1.3, 9.8, 3.1, 5.4–9.4 years for types E9, E11, E30 and EV-A71, respectively [26,27,28,57]), but the order of magnitude was almost the same. Therefore, noroviruses recombine about as frequently as human enteroviruses, which belong to the same taxonomic order (*Picornavirales*) and are similar to noroviruses in terms of global prevalence, transmission routes, replication sites and genetic diversity.

Recombination between structural and nonstructural genomic regions was detected within all analyzed norovirus genogroups. Both moderately related viruses with nucleotide distances less than 20% and more divergent viruses with nucleotide sequence distances up to 30–40% were involved in recombination (Figure 4A). Thus, the structural and nonstructural proteins of quite divergent noroviruses are compatible with each other, and co-infection of one cell with such viruses occurs regularly enough to provide the observed recombination frequency. No recombination was observed between noroviruses differing by more than 30% in nucleotide sequence in ORF1 and more than 40% in ORF2/3. Thus, noroviruses are characterized by complete recombination promiscuity, but only up to a certain level of genetic distance, which corresponds to a genogroup cut-off. Only a few inter-genogroup recombination events were reported previously [10,11,12,13]. None of them were reproduced here, because only partial genomic sequences were used in these previous studies. Several inter-genogroup recombination events were detectable by RDP4 methods in our dataset. However, none of them were recent (included genome fragments with distances below 45% in at least one genome region) and none were visible in PDC plots. Thus, there is no evidence that such recombination occurs systematically, as within genogroups, and it can be suggested that these were ancient recombination events that took place before the corresponding viruses diverged into distinct genogroups, and, strictly speaking, were not “inter-genogroup” recombination events. It may be speculated that ORFs from more distantly related viruses are not compatible, because such recombination does not occur at all. Therefore, genogroups are not merely man-made taxonomic units, but correspond to the biological properties of noroviruses. In terms of a ubiquitous lateral gene flow within and highly restricted between them (effectively a reproductive isolation), genogroups correspond to species in classical biology.

Almost all recombination events involved viruses infecting the same host, and only one was found between noroviruses infecting distinct host species. This suggests that host specificity of noroviruses is strict, and hypothetical “dead-end” cross-species spillover events that did not lead to establishment of a virus in a new host but could make its genetic material available to viruses of a new host, almost did not occur.

Due to the complexity of cell culture studies, there was no experimental work on recombination mechanisms in noroviruses. The first hypothetical explanation for recombination at the ORF1-ORF2 junction was a template switch facilitated by secondary RNA structure and sequence homology [64]. However, recent data challenge this explanation, at least as a unique one [9]. It has been well known that recombination in noroviruses occurs at the ORF1/2 junction, and that other recombinants occasionally emerge. An in vivo experimental study found that recombinants within ORF1 were generated in noroviruses, but less commonly than at the ORF1/2 junction by more than an order of magnitude [24]. It has been suggested that a highly conservative sequence (low sequence entropy) at the ORF1/2 overlap facilitates recombination [64,65]. PDC plots did not reveal massive recombination between very closely related noroviruses (over 90% nt sequence identity) within ORF1 or ORF2-3 (Figure 4B,C) as compared to frequent recombination at the ORF1/2 junction (Figure 4A). Thus, there was no obvious dependence between genetic similarity and recombination. This observation contradicts two hypotheses: one that sequence identity is required for a template switch by RNA polymerase, and the second, that protein compatibility limits survival of recombinants. Additionally, this observation suggests that virus-independent non-replicative recombination mechanisms facilitated by cellular RNA metabolism system that were described recently [66,67] are not likely to be dominant in noroviruses, because they are not known to be dependent on RNA properties or similarity and would not explain the ORF1/2 hot-spot. This leaves RNA secondary structure facilitating recombination [24] as a standing explanation, which would be compatible with explanation of recombination hot-spots in a distantly related poliovirus [68,69]. It is unlikely, however, that just any secondary structure would suffice, because the norovirus genome is almost evenly structured, and the structure pattern varies between isolates [70]. Certainly, further experimental studies are required.

The overall pattern of recombination in noroviruses (common events at the junction between genes encoding structural and nonstructural proteins) was similar to that in picornaviruses [71] and many other RNA viruses [72]. The temporal dynamics of recombination was also similar in enteroviruses and noroviruses that share the principal transmission route and belong to the same taxonomic order. However, there were important differences. In enteroviruses and other picornaviruses there is little apparent recombination in the structural proteins-encoding genome region (P1), but promiscuous recombination within species in the nonstructural proteins-encoding genome region (P2–P3) [71]. This contrasts with very limited recombination within the norovirus ORF1. While the structure of enterovirus genome in the biosphere may be seen as a cloud of promiscuously recombining nonstructural genome fragments and a set of more stable capsid genes, the norovirus genome may be viewed as a set of relatively more stable “Lego blocks” that are routinely shuffled within a genogroup.

## Data Availability

The data presented in this study are openly available at https://github.com/orlovartem/NoV_recombination (accessed on 27 November 2022).

## Supplementary Materials

The following supporting information can be downloaded at: https://www.mdpi.com/article/10.3390/v15020372/s1, Table S1: Intergenogroup recombination events in noroviruses detected by RDP4 in this study.
