# Supplementary material for: Patterns and Temporal Dynamics of Natural Recombination in Noroviruses
Source: Viruses. 2023 Jan 28;15(2):372. doi: 10.3390/v15020372 (PMC9961210; doi:10.3390/v15020372)
Supplement: Supplementary file 1 [file viruses-15-00372-s001.zip › Table S1.pdf]

Table S1. Intergenogroup recombination events in NoVs detected by RDP4 in this study.

Start of Recombinant Fragment	End of Recombinant Fragment	Length of Recombinant Fragment	Number of Recombination Events	Number of RDP4 Methods Supported the Event	Recombinant = Minor Parent + Major Parent (VP1 Genotype and P-Type)	Breakpoints within NoV Genome
5065	7419	2355	4	6	GIV.NA1_GIV.PNA1= GIV.2_GIV.P1+GNA2_GNA2.P1	ORF1/2 junction
3982	4120	139	17	5	GIII.2_GIII.P2= unknown+GI.7_GI.PNA2	ORF1
4482	4902	421	13	4	GVIII.1 _GII.P28= GI.2_GI.P2+GNA2_GNA2.P1	ORF1
5964	6778	815	2	4	GNA2_GNA2.P1= unknown+GIV.2_GIV.P1	ORF1/2, ORF2/3 junction
5833	6534	702	8	5	GVIII.1 _GII.P28= unknown+GII.5_GII.P5	ORF2
3652	3756	105	306	4	GII.4_GII.P31= GI.3_GI.P13+GII.4_GII.P31	ORF1
7290	18	142	37	4	GIX.1_GII.P15= unknown+GII.20_GII.P20	ORF3
6008	6652	645	161	4	GII.20_GII.P20= GII.4_GII.P31+GIX.1_GII.P15	ORF2
7208	40	224	18	4	GVIII.1 _GII.P28= unknown+GII.2_GII.P2	ORF3
4814	5830	1017	6	4	GVIII.1 _GII.P28= unknown+GII.12_GII.P16	ORF1, ORF2
